# *Zingiberaceae* extracts for pain: a systematic review and meta-analysis

**DOI:** 10.1186/s12937-015-0038-8

**Published:** 2015-05-14

**Authors:** Shaheen E. Lakhan, Christopher T. Ford, Deborah Tepper

**Affiliations:** 1 Global Neuroscience Initiative Foundation, Los Angeles, California USA; 2 Department of Musculoskeletal Biology, Institute of Ageing and Chronic Disease, The University of Liverpool, Liverpool, UK; 3 Neurological Institute, Cleveland Clinic, Cleveland, Ohio USA

**Keywords:** VAS, Ginger, Turmeric, Galangal, Curcumin, Curcuma

## Abstract

**Background:**

Members of the family Zingiberaceae including turmeric, ginger, Javanese ginger, and galangal have been used for centuries in traditional medicine. Preclinical studies of Zingiberaceae extracts have shown analgesic properties. This study aims to systematically review and meta-analyze whether extracts from *Zingiberaceae* are clinically effective hypoalgesic agents.

**Methods:**

Literature was screened from electronic databases using the key words *Zingiberaceae* AND pain OR visual analogue score (VAS) to identify randomized trials. From this search, 18 studies were identified, and of these, 8 randomized, double-blinded, placebo-controlled trials were found that measured pain by VAS for inclusion in the meta-analysis.

**Results:**

Findings indicated significant efficacy of *Zingiberaceae* extracts in reducing subjective chronic pain (SMD − 0.67; 95 % CI − 1.13 to − 0.21; *P* = 0.004). A linear dose-effect relationship was apparent between studies (R^2^ = 0.71). All studies included in the systematic review reported a good safety profile for extracts, without the renal risks associated with non-steroidal anti-inflammatory drugs, and with similar effectiveness.

**Conclusion:**

Our findings indicated that *Zingiberaceae* extracts are clinically effective hypoalgesic agents and the available data show a better safety profile than non-steroidal anti-inflammatory drugs. However, both non-steroidal anti-inflammatory drugs and *Zingiberaceae* have been associated with a heightened bleeding risk, and there have been no comparator trials of this risk. Further clinical studies are recommended to identify the most effective type of *Zingiberaceae* extract and rigorously compare safety, including bleeding risk.

## Background

Turmeric (*Curcuma longa*), ginger (*Zingiber officinale)*, Javanese ginger (*Curcuma zanthorrhiza*), and galangal (*Alpinia galanga*), which are members of the family *Zingiberaceae*, have been used for centuries in traditional medicine [1, 2]. *Zingiberaceae* have received scientific interest as dietary anti-inflammatory agents [3, 4], and pilot human trials have indicated benefits for chronic diseases including osteoarthritis, rheumatoid arthritis, and major depressive disorder [5–7]. Commonly prescribed medications for pain, principally non-steroidal anti-inflammatory drugs (NSAIDs), have side effects with long-term use [8], leading to calls for safer tools for the management of chronic pain [9].

Turmeric’s principal bioactive is the polyphenol curcumin [10], which can be extracted using organic solvents [11]. Curcumin has anti-inflammatory properties [12]; its mechanisms include down-regulation of nuclear factor (NF)-κB [13] and cyclooxygenase 2 (Cox-2) [14]. Animal studies have shown antinociceptive effects of oral curcumin [15] and indicated the involvement of ATP-sensitive potassium channels [16]. Pilot human studies of curcumin have shown promise for improving symptoms of rheumatoid arthritis and inflammatory bowel disease [5, 17]. Curcumin is poorly bioavailable and it is likely that its benefits are mediated via secondary metabolites [18].

The primary anti-inflammatory components of ginger are gingerol and zingerone [3, 19]; however, other ginger components also have anti-inflammatory activities *in vivo* [20]. Mechanisms of action include modulation of leukotriene and prostaglandin synthesis, and inhibition of NF-κB [3, 19, 21]. One meta-analysis was previously conducted on ginger for pain, but the authors concluded too few rigorous studies were available at that time to make a confident recommendation [22].

Galangal polyphenols were shown to protect against lipopolysaccharide-induced injury in a rodent model, indicating a functional anti-inflammatory effect *in vivo* [23, 24]. *In vitro* work has suggested that the galangal component 1′-acetoxychavicol modulates a transient receptor potential (TRP) cation channel [25]. Javanese ginger contains curcumin [26] and zanthorrhizol, which is also a Cox-2 inhibitor [27]. Javanese ginger extract and zanthorrhizol have anti-inflammatory effects *in vivo* [28] and Javanese ginger extract displayed anti-nociceptive effects in mice [29].

Preclinical studies of *Zingiberaceae* extracts have shown antinociceptive effects [29–32]. Previous reviews have considered *Zingiberaceae* species for particular clinical situations [33, 34], but no prior reviews have examined all *Zingiberaceae* for chronic pain. The present review and meta-analysis aims to include randomized controlled trials (RCTs) that have used *Zingiberaceae* extracts for a condition involving chronic pain. Since the time window for absorption and action of *Zingiberaceae* extracts is unknown, but is probably less than 24 h based on data for other herbal extracts [35], we only included studies involving treatment periods lasting 24 h or longer. While chronic pain is typically defined as pain present for at least 3 months, for this meta-analysis we included pain present for greater than 24 h to capture a sufficient number of studies of non-acute pain.

The aim of this systematic review and meta-analysis is to assess the potential for hypoalgesic effects of *Zingiberaceae* extracts in conditions involving chronic pain.

## Methods

### Literature search strategy

The meta-analysis was conducted according to Cochrane and PRISMA guidelines [36]. Published studies were identified by searching PubMed, ScienceDirect, and Cochrane Library databases, using the following search terms, which include the common and taxonomic names of the *Zingiberaceae* species, and specific bioactive components found in each plant: turmeric, curcumin, ginger, galangal, *Zingiberaceae*, *Curcuma*, *Zingiber, Kaempferia, Alpinia,* curcuminoid, turmerone, gingerol, shogaol, zingiberene, zingiberol, zingerone, curcumene, galangin, zanthorrhizol, AND pain OR visual analogue score OR VAS. The references within each identified report were manually reviewed. Publications were included that were published before December 2014.

### Selection of studies for inclusion in the systematic review

Studies were included in the systematic review if they met the following criteria: **a)** the study was randomized; **b)** the study included a patient group with chronic pain, i.e., pain lasting 24 h or longer; **c)** extracts from one or more *Zingiberaceae* species were given either alone or in combination with agents intended to increase the bioavailability of the *Zingiberaceae* bioactives, but not together with other treatments or non-*Zingiberaceae* herbal extracts; **d)***Zingiberaceae* extracts were given for 24 h or longer, and **e)** pain was measured as a study outcome. Papers were excluded before review stage if they were not peer-reviewed, full-length, original research reports (for example they were reviews, editorials, conference abstracts, etc.)

### Selection of studies for inclusion in the meta-analysis

Further selection criteria were applied for inclusion of papers into the meta-analysis. Papers were excluded from the meta-analysis that were: **a)** not placebo-controlled; **b)** not double-blinded; or **c)** did not measure pain using VAS.

### Data extraction

Group sample sizes, and means and standard deviations for pain, were extracted from each included report for pooled analysis. To focus the meta-analysis and maximize the accuracy of comparisons between studies, we chose to only extract and analyze data for the VAS measurement of pain. Other measurements varied widely between the included papers, which investigated divergent patient groups. Where studies involved more than one treatment group, data was only extracted for the group treated with the *Zingiberaceae* extract in its least processed and/or highest dose form. For studies reporting results for multiple time points, data were extracted for the longest treatment time period, except in the study by Black et al. [37], where, because pain in the placebo group decreased over time after the eccentric exercise protocol, the earliest time point of 24 h supplementation was selected.

### Data analysis

The standardized mean difference (SMD) was calculated allowing for a continuous pain rating scale such as the VAS. The SMD is a useful statistical tool when studies all assess the same outcome but measure it in a variety of ways. Effect sizes for all included studies were calculated and weighted by variance. In accordance with Higgins et al., 2003 [38], heterogeneity was considered to be low at I^2^ ≤ 25 %, moderate at I^2^ ≤ 50 %, high at I^2^ ≤ 75 %, and very high at I^2^ > 75 %. All calculations were performed using the StatsDirect software package, version 2.7.9. P-values were considered significant at ≤0.05. The potential for publication bias was assessed using the funnel plot method [39]. A symmetric funnel plot indicates that there was little bias based on the sample size of the analyzed studies, whereas an asymmetric funnel plot suggests that a meta-analysis may be flawed because of inappropriate weight given to small versus large studies. We have no specific reason to suspect bias in this research area. Since the included studies for quantitative analysis used a wide range of *Zingiberaceae* extract doses, a post-hoc decision was made to draw a dose-effect curve for SMDs against mg of extract/day used in each study.

## Results

**Fig. 1:**
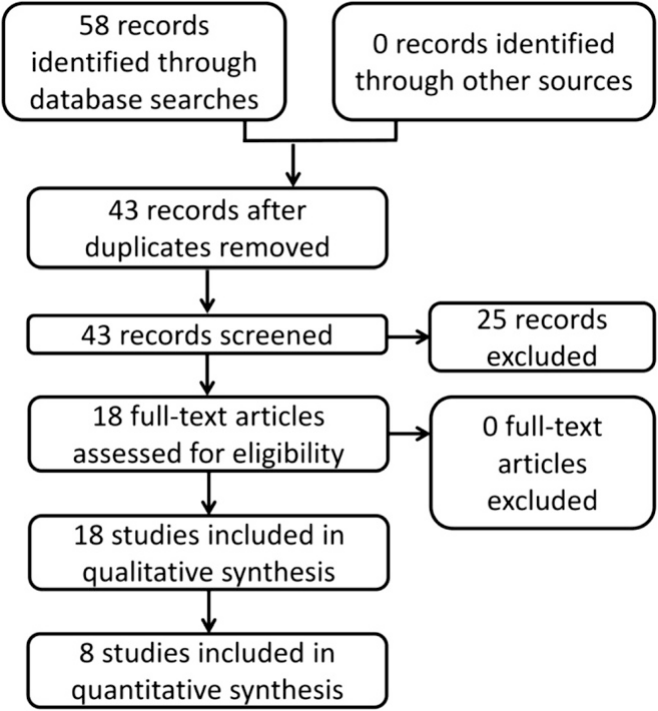
PRISMA flow chart showing the numbers of identified, screened, included, and excluded studies for the systematic review (qualitative synthesis) and meta-analysis (quantitative analysis)

We screened 43 unique records and rejected 25 of these because either they were not controlled or they investigated pain lasting for less than 24 h. The systematic review included 18 randomized trials for qualitative analysis and the meta-analysis included 8 RCTs (Fig. 1). Those 10 RCTs that were included in the qualitative synthesis, but not the quantitative analysis, were excluded from the meta-analysis either because they were not double-blinded, or they were not placebo-controlled, or they measured pain by a method other than VAS.

### Systematic review

Chronic pain has traditionally been described as pain present for 12 weeks or longer, although this definition has been shifting to include a less rigid time frame. Chronic pain as defined by the International Association for the Study of Pain (IASP) as “pain that persists beyond normal tissue healing time”. This meta-analysis utilized a broad definition of chronic pain to include pain greater than 24 h in order to capture a meaningful number of studies.

Chronic pain is a symptom of many long-term medical conditions, including cancer, arthritis, nerve damage after traumatic injury, and neuropathy associated with chronic conditions such as diabetes. It has serious adverse effects on multiple dimensions of quality of life [40]. In Europe, 19 % of adults were found to experience chronic pain of moderate to severe intensity in a survey published in 2006, and of the survey respondents, 40 % felt that management of their pain was inadequate [41]. Side effects of currently prescribed treatments for chronic pain have led to calls for the development of new and safer approaches [8]. This systematic review explores randomized human trials of *Zingiberaceae* extracts for chronic pain, defined for this meta-analysis as pain lasting for longer than 24 h.

### Chronic pain in arthritic conditions

The first randomized trial of *Zingiberaceae* extracts that measured pain as an outcome investigated the effects of 510 mg of ginger extract/day for osteoarthritis using a crossover, double-blinded, placebo-controlled study design. The ginger-treated group reported significantly less pain compared with the placebo group for the first period before crossover, but not after crossover, while the ibuprofen group showed a stronger reduction in pain [42]. Shortly thereafter, another double-blinded, randomized, placebo-controlled trial was conducted for osteoarthritis, again with a treatment dose of 510 mg/day, but using a mixed *Zingiberaceae* treatment comprising ginger and galangal extracts. A moderate reduction of pain on walking was detected after 6 weeks of treatment, compared with the placebo group [43]. A 6-month, placebo-controlled study in symptomatic gonarthritis (knee arthritis) patients investigated the effects of a higher dose of 1000 mg/day of ginger extract with crossover between ginger and placebo groups at 3 months. In the second period after crossover, significantly less pain was reported in the ginger group, compared with the placebo group [44]. Ginger extract was used in a further study for osteoarthritis at 340 mg/day dosage, and was found to be equally as effective as diclofenac for pain relief, but with a superior safety profile, indicated by significantly fewer adverse gastropathic changes in the ginger group, compared with the diclofenac group [45]. A trial of curcumin for knee osteoarthritis, in which patients took 1000 mg curcumin/day as an adjuvant to diclofenac treatment, found no significant difference between the curcumin + diclofenac group and the placebo + diclofenac group [46]. An open-label comparative trial treated patients with knee osteoarthritis using diclofenac + placebo, ginger extract (1500 mg/day) + placebo, or diclofenac + ginger. The results showed that all three treatment groups reported significantly less pain after 12 weeks of treatment than at baseline, and the diclofenac plus ginger group reported significantly less pain than the diclofenac + placebo and ginger + placebo treatment groups [47]. Another trial in knee osteoarthritis, investigating the effects of supplementation with 1000 mg/day of turmeric extract, found significantly less pain in the treatment group compared with the placebo group [48]. A trial comparing 1500 mg/day of turmeric extract with ibuprofen for knee osteoarthritis concluded that turmeric extract and ibuprofen were equally effective in reducing pain [49]. In a different study, treatment with 1500 mg/day of curcuminoids was found to significantly reduce pain in knee osteoarthritis compared with a placebo group [50]. In summary, there have been multiple randomized trials of *Zingiberaceae* extracts for arthritis (osteoarthritis and gonarthritis), which have mostly found moderate reductions in reported subjective pain, with *Zingiberaceae* extracts showing comparable effectiveness to standard NSAID treatments. The evidence for turmeric extracts/curcumin for chronic pain in osteoarthritis is stronger than that for ginger extracts.

### Pain in primary dysmenorrhea

A trial of ginger extract for primary dysmenorrhea found that 1000 mg of ginger extract/day resulted in a reduction of pain with equal effectiveness to each of two NSAID control groups (mefenamic acid and ibuprofen) [51]. A placebo-controlled trial was conducted, with patients taking 1500 mg of ginger extract/day. Significant reductions in the severity and duration of pain were detected in the ginger-treated group, compared with the placebo group [52]. Although only two randomized studies were available, their findings suggest that ginger extract may be an effective treatment for pain in primary dysmenorrhea.

### Pain in delayed onset muscle soreness

Volunteers who performed a strenuous eccentric exercise protocol (18 eccentric extensions of the elbow flexor muscles against resistance at 120 % of their concentric one-repetition maximum) and were treated with either raw or heat-treated ginger extracts at 2000 mg/day dosage reported less pain associated with delayed onset muscle soreness than a placebo group [36]. A similar study was performed using a high-bioavailability curcumin formulation, with 400 mg/day curcumin given to volunteers who underwent a milder eccentric exercise protocol (downhill running on a treadmill for 45 min). This study also found a reduction in pain for the treated group, compared with the placebo group, however this difference was significant only for the muscles of the anterior thigh [53]. These studies suggest that *Zingiberaceae* extracts may have a moderate hypoalgesic effect for delayed onset muscle soreness following eccentric exercise. Further trials are recommended to determine the most effective extract type and dose.

### Pain in recovery from surgery

A double-blinded, placebo controlled trial of high-dose curcumin (2000 mg/day) for patients recovering from laparoscopic cholecystectomy found a significant reduction in pain at weeks 1 and 3 of treatment in the treated group, compared with the placebo group [54]. The results of this single study suggest curcumin may alleviate pain following surgery.

### Chronic pain in irritable bowel syndrome

A pilot study of turmeric extract for irritable bowel syndrome (IBS), which had two dose groups, both with relatively low intake (72 and 144 mg/day, respectively), found a modest but significant reduction in abdominal pain for both the lower- and higher-dose turmeric extract treatment groups [55]. Another trial investigated Javanese ginger extract for IBS, with patients taking 60 mg of extract/day for 18 weeks in a double-blinded, placebo-controlled study. In this trial, no significant difference in pain was found between the treatment and placebo groups [56]. In light of findings for *Zingiberaceae* extracts for other conditions, we recommend further trials in IBS using higher treatment doses.

### Pain in cancer radiotherapy-induced dermatitis

A double-blinded, placebo controlled trial of breast cancer patients undergoing radiotherapy investigated the effects of very high-dose curcumin (6000 mg/day) on pain associated with radiation dermatitis. Although significant improvement was seen for some other radiation dermatitis symptoms with curcumin treatment, no significant difference was seen for pain scores between the curcumin-treated and placebo groups [57]. Based on this single study, curcumin cannot be recommended as a pain management tool for radiation dermatitis.

### Meta-analysis

The studies that were determined to be eligible for inclusion in the quantitative analysis included five studies using ginger extracts, two studies using curcuminoids or curcumin, and one study using Javanese ginger extract. The patient groups included four groups with arthritis (osteoarthritis or gonarthritis), one group with IBS, one group with primary dysmenorrhea, one group recovering from surgery, and one group with muscle soreness following eccentric exercise. All studies included adult participants at least 18 years of age. Table 1 lists the methods and results of each included study, organized by date of publication. In all included studies, the majority of subjects were female, and in one study, all of the subjects were female. While this could be interpreted to imply bias in sampling or selection, it can also be interpreted as being due to the known higher risk of females for arthritis [58], IBS [59], and gallstones necessitating laparoscopic cholecystectomy [60].

**Table 1:** Characteristics of included studies for meta-analysis

Study	Patient group	Number of subjects		Percent female		Mean age ± SD (years)		Intervention	Control	Results for pain
		Treated	Control	Treated	Control	Treated	Control			
**Bliddal et al., 2000** [42]	Osteoarthritis of knee or hip.	56	56	73	73	Age range 24–87	Age range 24–87	Capsules containing 170 mg ginger extract, 3 capsules per day for 3 weeks.	Placebo *(crossover design)*.	Pain was not significantly different between the ginger and placebo groups.
**Altman et al. 2001** [43]	Osteoarthritis of knee.	124	123	60	63	64.0 ± 11.5	66.3 ± 11.6	Capsules containing 255 mg of mixed ginger and galangal rhizome extracts, 2 capsules per day for 6 weeks.	Placebo *(parallel design)*.	Pain on walking was significantly lower in the ginger/galangal group after 6 weeks of treatment compared with the placebo group.
**Wigler et al., 2003** [44]	Symptomatic gonarthritis.	29	29	79	79	Age range 42–85	Age range 42–85	Capsules containing 250 mg ginger rhizome extract, 4 capsules per day for 3 months.	Placebo *(crossover design*).	Pain was significantly lower in the ginger group compared with the placebo group.
**Brinkhaus et al., 2005** [56]	Irritable bowel syndrome.	24	58	58	62	49.5 ± 14.5	49.0 ± 9.1	Capsules containing 20 mg Javanese ginger extract, 3 capsules per day for 18 weeks.	Placebo *(parallel design)*.	Pain was not significantly different between the Javanese ginger and placebo groups.
**Black et al., 2010** [37]	Muscle soreness following exercise.	20	20	65	65	20.6 ± 0.6	21.4 ± 0.8	Capsules containing 333 mg ginger rhizome extract, 6 capsules per day for 3 days following eccentric exercise.	Placebo *(parallel design)*.	Pain was significantly lower in the ginger group compared with the placebo group.
**Agarwal et al., 2011** [54]	Recovering from surgery.	25	25	84	80	38.4 ± 12.8	37.2 ± 12.7	Capsules containing 500 mg curcumin, 4 capsules per day for 3 weeks.	Placebo *(parallel design)*.	Pain was significantly lower in the curcumin group compared with the placebo group.
**Rahnama et al., 2012** [52]	Primary dysmenorrhea.	59	46	100	100	21.4 ± 2.0	21.3 ± 2.2	Capsules containing 500 mg ginger rhizome powder, 3 capsules per day for 5 days.	Placebo *(parallel design)*.	Pain was significantly lower in the ginger group compared with the placebo group.
**Panahi et al., 2014** [50]	Osteoarthritis of the knee.	19	21	73	81	57.32 ± 8.78	57.57 ± 9.05	Capsules containing 500 mg curcuminoids and 5 mg black pepper extract (bioavailability enhancer), 3 capsules per day for 6 weeks.	Placebo *(parallel design)*.	Pain was significantly more reduced in the curcuminoid treatment group, compared with the placebo group.

#### Publication bias

**Fig. 2:**
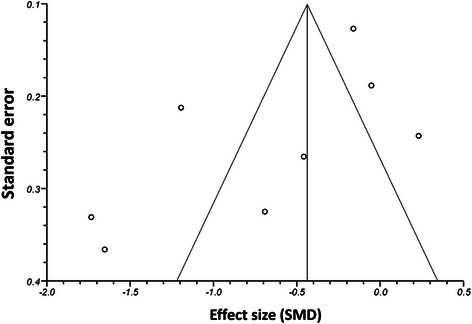
A funnel plot for pain, showing standard error plotted against effect size (SMD). Each data point represents one RCT included in the meta-analysis. The plot was not completely symmetrical, indicating potential bias, but bias could not be concluded due to a lack of statistical significance (Egger’s test, P = 0.10). A high degree of inconsistency between studies (I^2^ = 87.5) may contribute to the asymmetry of the funnel plot

Following the methods of Egger et al., 1997 [39], a funnel plot was drawn for standard error against effect size (Fig. 2) to assess the possibility of publication bias, for example, the selective reporting of trials showing positive results, but not those producing negative results in the literature. The funnel plot was not fully symmetrical, which is an indicator for possible bias, but bias could not be concluded due to lack of statistical significance (Egger’s test, *P* = 0.10; a P-value less than 0.05 would demonstrate significant bias). In addition, inconsistency between studies was very high (I^2^ = 87.5 %), a factor that can contribute to asymmetry in funnel plots without necessarily implying bias.

#### Meta-analysis of Zingiberaceae extracts for pain

**Fig. 3:**
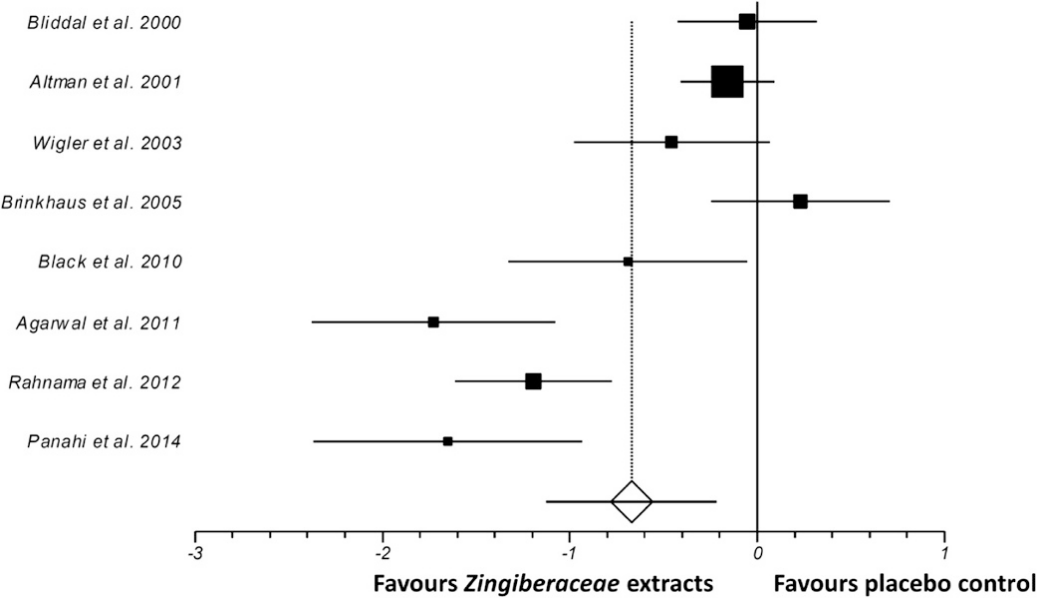
Random-effects meta-analysis Forest plot showing standardized mean differences (SMDs) for each study of *Zingiberaceae* extracts included in the quantitative analysis, sorted by date of publication. The meta-analysis findings indicated that *Zingiberaceae* extracts significantly reduced subjective pain (P = 0.004; SMD -0.67; 95 % CI -1.13 to -0.21). Earlier studies tended to use lower doses, which may explain the tendency for more recent studies to have larger effect sizes. Each study is represented by a square, with the area of the square corresponding to the weight given to that study in the meta analysis (weighting based on the number of subjects in each trial). The estimated overall effect size is displayed as a diamond. Horizontal lines show 95 % confidence intervals

One of the inclusion criteria by which the 8 RCTs of *Zingiberaceae* extracts included in the meta-analysis were selected was that they all used the same measurement tool (VAS) for the assessment of pain. A random-effects meta-analysis was performed according to the methods of DerSimonian and Laird, 1986 [61] (Fig. 3). This was necessary because of the heterogeneity of effects in the included studies. This analysis decreased the heterogeneity and allows for meaningful therapeutic findings. The standardized mean difference (SMD) for pain was large (−0.67, 95 % CI: −1.13 to −0.21), with significantly lower reported subjective pain in the experimental group compared with the control group (*P* = 0.004). Heterogeneity between studies was very high (I^2^ = 87.5 %). SMDs and confidence intervals for studies included in the meta-analysis are shown in Table 2.

**Table 2:** Effect sizes (SMDs) and confidence intervals for each study included in the meta-analysis. Negative SMDs show a reduction of pain in the treatment group for that study compared with the placebo control group

Study	N (treatment)	N (control)	SMD	95 % CI low	95 % CI high
**Bliddal et al. 2000** [42]	56	56	−0.05	−0.42	−0.32
**Altman et al. 2001** [43]	124	123	−0.16	−0.41	0.09
**Wigler et al. 2003** [44]	29	29	−0.46	−0.98	0.07
**Brinkhaus et al. 2005** [56]	24	58	0.23	−0.24	0.71
**Black et al. 2010** [37]	20	20	−0.69	−1.33	−0.05
**Agarwal et al. 2011** [54]	25	25	−1.73	−2.38	−1.08
**Rahnama et al. 2012** [52]	59	46	−1.19	−1.61	−0.77
**Panahi et al. 2014** [50]	19	21	−1.65	−2.37	−0.93

#### Dose-dependent effect of Zingiberaceae extracts for pain

**Fig. 4:**
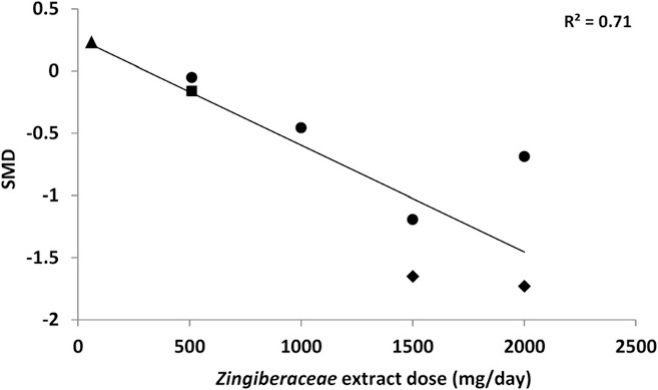
A scatter plot showing the effect size (standardized mean difference (SMD), y-axis) for each study included in the meta-analysis, plotted against the dose of *Zingiberaceae* extract that was used in that study (x-axis). The results show that studies using larger doses of *Zingiberaceae* extracts tended to report more effective hypoalgesia (R^2^ = 0.71). Different markers represent the extract type: Triangle = Javanese ginger; Circle = Ginger; Square = Mixed ginger and galangal; Diamond = Curcuminoids/curcumin

To determine whether the reduction in subjective pain by turmeric showed a dose-effect relationship across the 7 studies included in the quantitative meta-analysis, a scatter plot was drawn between the SMD for each study and the total dose of *Zingiberaceae* extract taken by subjects per day (Fig. 4). The results suggested a linear dose-effect relationship (R^2^ = 0.71).

## Discussion

Compared with placebo controls, an overall moderate to large effect of *Zingiberaceae* extracts (including turmeric, ginger, and Javanese ginger) was found for reported chronic pain. While there was substantial heterogeneity between studies in their effect sizes, this may partly be explained by the wide variability in the doses of *Zingiberaceae* extracts used in each study. A dose-response curve was plotted and revealed a strong dose-effect relationship.

No previous meta-analyses have considered all *Zingiberaceae* for chronic pain, but one prior analysis was conducted on ginger extracts only. The conclusion of that study was that there were insufficient studies on ginger at that time to make any recommendation on the effectiveness of ginger extracts for pain [22]. Meta-analyses have been conducted on *Zingiberaceae* extracts for several other parameters. A meta-analysis of curcumin for blood lipid levels did not find any significant effects [62]. A meta-analysis of ginger extracts for chemotherapy-induced nausea and vomiting did not identify any significant benefit compared with placebo [63], but two other meta-analyses of ginger for the treatment of nausea and vomiting induced by pregnancy [64] and during post-operative recovery [65], respectively, found significant improvement of symptoms by ginger extract treatment compared with placebo controls.

Our results indicate that *Zingiberaceae* extracts may be a useful tool for clinical pain management. However, certain side effects have been reported that may limit the use of *Zingiberaceae* extracts for specific patient groups. Allergic reaction to *Zingiberaceae* extracts occurs rarely [66]. Rodent studies have indicated the potential for *Zingiberaceae* extracts to cause hepatotoxicity [67], and so their use is cautioned in patients with existing liver dysfunction. In addition, *Zingiberaceae* extracts have been reported to have anticoagulant activities [68] and may potentially exacerbate clotting disorders [69]. Nausea (usually mild) is a reported potential effect of *Zingiberaceae* extracts; however, Drozdov et al., 2012 [46] reported a superior gastropathic safety profile for ginger extract compared with diclofenac treatment in osteoarthritis patients. The study included in our systematic review that used the highest dose, with patients taking 6000 mg/day of curcumin for 4-7 weeks, reported no significant adverse events and no significant increase in gastropathy (diarrhea) in the curcumin group compared with the placebo group [57].

### Study limitations

This meta-analysis has several limitations. One is the inclusion of only a small number of studies (8 RCTs); although those studies that were included met very rigorous criteria, all of the included studies were randomized, double-blinded, placebo-controlled, and each used the same measurement tool (VAS) to quantify subjective pain. Another limitation is that the included studies showed very high heterogeneity. This variability is, however, likely to be at least partly due to the strong dose-effect relationship that we identified and the wide range of doses used among the studies under analysis (60-2000 mg of extract/day). The study of different *Zingiberaceae* species and patient populations in each trial may also contribute to the heterogeneity. The number of included studies was too small to perform subgroup analysis. A final limitation is that the included studies showed a strong gender skew towards female subjects. Further study in male populations would help to confirm whether *Zingiberaceae* extracts offer uniform pain relief across genders, although there is no clear biological reason to suspect any difference between genders in the response to *Zingiberaceae* extracts.

Although the studies included in the meta-analysis did not report significant adverse events from *Zingiberaceae* treatment, there are indications that caution needs to be exercised in recommending treatment without further studies. *Zingiberaceae* extracts are generally regarded as safe in lower dosages of concentrated extracts [18, 69]. At higher dosages, there may be an increased risk of bleeding secondary to impairment of the clotting cascade or platelet dysfunction [68, 70], although this risk is not well defined. Turmeric supplementation has been associated with oxalate kidney stones [71]. A toxicology analysis of curcumin and its derivatives showed there can be a hepatotoxic effect that is dose-related [72]. Ginger and turmeric have an antiplatelet effect, and coupling either of them with NSAIDs can increase the risk of significant bleeding [73]. The degree to which these supplements may increase bleeding risk is not well known, however it is recommended that they be stopped two weeks prior to any surgery or significant dental procedure [74].

## Conclusions

Chronic pain is a symptom of numerous medical disorders that may severely reduce quality of life, and its management is a core requirement of clinical care. Drugs commonly used for the relief of chronic pain have side effects that may interdict their use. Our results show that *Zingiberaceae* rhizome extracts, which have a good record of safety in human studies, offer effective relief from chronic pain. The dose-dependent effect identified implies that higher doses may potentially increase the effect further. The caveat to these findings is that they are based on a relatively small number of RCTs (8 studies), though these studies were included according to rigorous criteria. Long term and larger safety studies of these supplements, particularly at higher doses, are suggested. In summary, the available evidence suggests that *Zingiberaceae* extracts have hypoalgesic effects for chronic pain. We recommend further study of high-dose *Zingiberaceae* extracts for chronic pain management in clinical practice, and we also encourage focused assessment to determine the most effective *Zingiberaceae* species, extract doses, and bioactive chemicals; such research may potentially facilitate the development of higher potency hypoalgesic agents based on *Zingiberaceae* bioactives.
